# Altered Levels of Sphingosine, Sphinganine and Their Ceramides in Atopic Dermatitis Are Related to Skin Barrier Function, Disease Severity and Local Cytokine Milieu

**DOI:** 10.3390/ijms21061958

**Published:** 2020-03-13

**Authors:** Ruzica Jurakic Toncic, Ivone Jakasa, Suzana Ljubojevic Hadzavdic, Susan MI Goorden, Karen JM Ghauharali-van der Vlugt, Femke S Stet, Anamaria Balic, Mikela Petkovic, Borna Pavicic, Kristina Zuzul, Branka Marinovic, Sanja Kezic

**Affiliations:** 1 Department of Dermatology and Venereology, University Hospital Center, Zagreb and University of Zagreb School of Medicine, 10000 Zagreb, Croatia; rjtoncic@gmail.com (R.J.T.); suzana.ljubojevic@gmail.com (S.L.H.); jovicanamaria@gmail.com (A.B.); dr.mikela@gmail.com (M.P.); borna.pavicic@gmail.com (B.P.); zuzulkristina@gmail.com (K.Z.);; 2 Laboratory for Analytical Chemistry, Department of Chemistry and Biochemistry, Faculty of Food Technology and Biotechnology, University of Zagreb, 10000 Zagreb, Croatia or; 3 Amsterdam UMC, University of Amsterdam, Coronel Institute of Occupational Health, Amsterdam Public Health research institute, 1105 AZ Amsterdam, The Netherlands; 4 Laboratory Genetic Metabolic Disease, Amsterdam UMC, 1105 AZ Amsterdam, The Netherlands

**Keywords:** atopic dermatitis, biomarkers, stratum corneum, ceramides, sphingosine, sphinganine

## Abstract

Dysfunctional skin barrier plays a key role in the pathophysiology of atopic dermatitis (AD), a common inflammatory skin disease. Altered composition of ceramides is regarded as a major cause of skin barrier dysfunction, however it is not clear whether these changes are intrinsic or initiated by inflammation and aberrant immune response in AD. This study investigated the levels of free sphingoid bases (SBs) sphingosine and sphinganine and their ceramides and glucosylceramide in the stratum corneum (SC) and related them to skin barrier function, disease severity and local cytokine milieu. Ceramides were measured in healthy skin, and lesional and non-lesional skin of AD patients by a novel method based on deacylation of ceramides which were subsequently determined as corresponding sphingoid bases by using liquid chromatography–tandem mass spectrometry (LC–MS/MS). The cytokine levels were determined by multiplex immunoassay. Atopic skin showed increased levels of most investigated markers, predominantly in lesional skin. The largest difference in respect to healthy skin was found for glucosylceramide with respective median values of 0.23 (IQR 0.18–0.61), 0.56 (IQR 0.32–0.76) and 19.32 (IQR 7.86–27.62) pmol/µg protein for healthy, non-lesional and lesional skin. The levels of investigated ceramide markers were correlated with disease severity (scoring atopic dermatitis, SCORAD) and skin barrier function (trans-epidermal water loss, TEWL) and furthermore with cytokines involved in innate, Th-1, and Th-2 immune response. Interestingly, the strongest association with SCORAD was found for sphinganine/sphingosine ratio (*r* = ‒0.69, *p* < 0.001; non-lesional skin), emphasizing the importance of SBs in AD. The highest correlation with TEWL was found for glucosylceramide (*r^2^* = 0.60, *p* < 0.001), which was investigated for the first time in AD. Findings that the changes in SBs and ceramide levels were predominant in lesional skin and their association with disease severity and cytokine levels suggest an immune-system driven effect. A novel analysis method demonstrates a robust and simple approach that might facilitate wider use of lipid biomarkers in the clinics e.g., to monitor (immune) therapy or dissect disease endotypes.

## 1. Introduction

**Figure 1:**
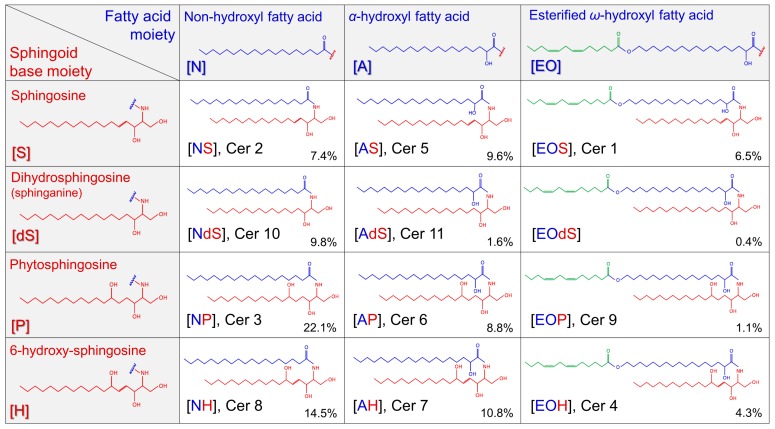
Main ceramide classes. The sphingoid bases consist of dihydrosphingosine (dS) (also referred to as sphinganine), sphingosine (S), phytosphingosine (P), or 6-hydroxy sphingosine (H). The fatty acid (FA) may be a non-hydroxyl with FA (N), α-hydroxyl fatty acid (A), or esterified ω-hydroxyl fatty acid (EO). In the figure next to class name, also old nomenclature for ceramides is given. In the right lower part of each ceramide class its relative amount is given as percentage of total ceramide amount [10].

Atopic dermatitis (AD) is a common inflammatory skin disease affecting up to 20% of children and 10% of adults [1]. AD is characterized by a dysfunction in the immune system, with a dominant helper T-cell T_H_2/T_H_22 skewing, and variable activation of T_H_17/T_H_1 subtypes [1,2]. Patients with AD have high prevalence of cutaneous infections attributed to abnormalities in the innate immune system, altered lipid biosynthesis, and reduced levels of antimicrobial proteins and epidermal protein filaggrin [3,4,5,6]. One of the major hallmarks, and an important etiological factor in AD is dysfunction of the skin barrier [7,8]. The complex crosstalk between the epithelial barrier, immunity and cutaneous microbiome has been recognized as a crucial step in AD etiology [1,2,3,4]. The epidermal permeability barrier is localized in the extracellular matrix composed of lamellae enriched in ceramides (CER), free fatty acids (FFAs), and cholesterol [9,10]. Major lipid components are CER, accounting for about 50% of total stratum corneum (SC) lipid mass [11]. CER are composed of a sphingoid base (SB), linked via an amide bond to a fatty acid (FA) [12,13,14]. There are four SBs, which in combination with a FA-chain give rise to 12 main ceramide classes (Figure 1) [10]. 

While older work in the field of ceramides in AD focused on the (relative) amount of ceramide classes, recent studies were related to changes in the acyl-chain length. As convincingly demonstrated, SC lipid lamellae in AD patients are enriched with ceramides with shorter chain length FA [15,16,17,18,19]. The relative amount of short FA correlated with changes in lipid organization and skin barrier function and was affected by disease severity [17]. Given the fact that there are hundreds of CER species in human SC [10,20], their determination is challenging hampering their wider application in the clinics e.g., to monitor therapy or dissect disease endotypes, which has become of crucial importance in the era of novel immunotherapies for AD. In the present study, we made an attempt to disentangle complexity by deacylation of ceramides (i.e., cutting the FA chain from the CER) and measuring the sum of all CER that share the same SB. As SBs are also measured before deacylation, this will provide not only information on the amount of CER but also of free SBs which are known to contribute to skin barrier, antimicrobial defense and regulation of cell proliferation, all of them known to be affected in AD [21,22,23]. We limited our study to sphingosine and sphinganine (dihydrosphingosine) comprised of 18 C-atoms as they are the most abundant SBs in human SC [10]. Their corresponding CER not only substantially constitute to the total CER content (Figure 1), but fulfill important roles in the maintenance of skin barrier function and cell signaling. Next, we will determine the levels of glucosylceramide, one of SC ceramide precursors. Degradation of glucosylceramide is regulated by β-glucosylcerebrosidase (GBA) which showed altered expression in AD skin [15,24]. 

The main aim of the present study is to investigate the relationship of the ceramide markers including free sphingoid bases sphinogosine (d18:1) and sphinganine (d18:0), their respective ceramides, CER (dS) and CER (S) and glucosylceramide (d18:1) with disease severity and skin barrier function, which might support the monitoring of disease course e.g., in clinical trials. As novel therapies in AD target immune response, we will furthermore explore the relationship between lipid markers and levels of a broad spectrum of immunological mediators.

## 2. Results

### 2.1. Biophysical Parameters

**Figure 2:**
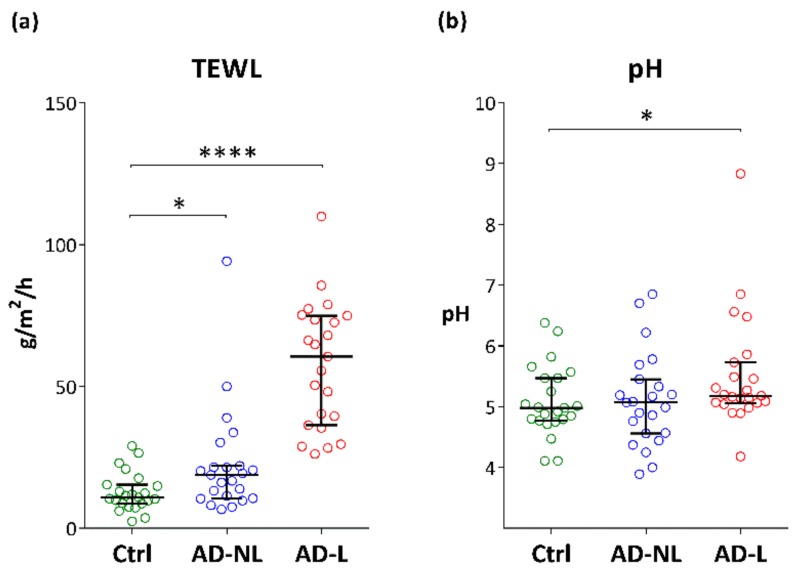
Trans-epidermal water loss (TEWL) (**a**) and pH (**b**) in healthy controls (Ctrl) and non-lesional (AD-NL) and lesional (AD-L) skin of AD patients. Data are shown as median with interquartile ranges. **** *p* < 0.0001, * *p* < 0.05.

The values of TEWL and pH measured in lesional and non-lesional skin in AD patients were significantly higher as compared to healthy skin (Figure 2a,b). This increase was more prominent in lesional skin.

### 2.2. Sphingoid Bases and Their Ceramides in Healthy and AD Skin

**Figure 3:**
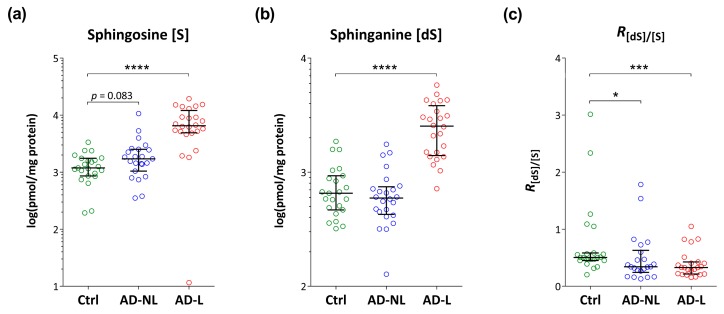
Levels of free sphingoid bases’ (**a**) sphingosine (S) and (**b**) sphinganine (dS), and (**c**) their ratio *R*_[dS]/[S]_ in the skin of healthy controls (Ctrl) and non-lesional (AD-NL) and lesional (AD-L) skin in AD patients. Data are shown as median with interquartile ranges. **** *p* < 0.0001, *** *p* < 0.001, * *p* < 0.05.

As compared to healthy skin, the levels of sphingosine and sphinganine are elevated in AD, but only in lesional skin (Figure 3a,b). The ratio of sphinganine over sphingosine showed a gradual decrease from healthy skin to respectively non-lesional and lesional AD skin (Figure 3c). Both lesional and non-lesional AD skin showed significantly lower values as compared to healthy skin.

**Figure 4:**
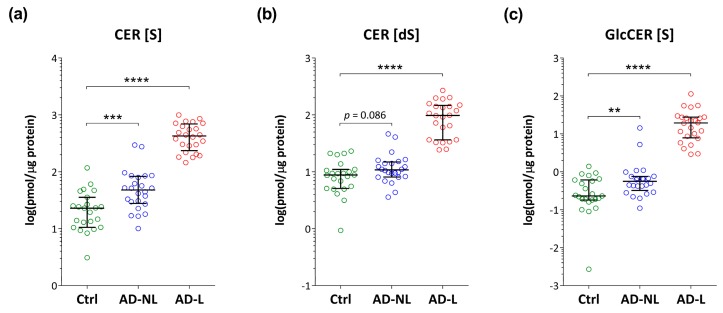
Levels of (**a**) sphingosine- and (**b**) sphinganine-based ceramides (respectively, CER (S) and CER (dS)) and (**c**) glucosylceramide (GlcCER (S)) in the skin of healthy controls (Ctrl) and non-lesional (AD-NL) and lesional (AD-L) skin in AD patients. Data are shown as median with interquartile ranges. **** *p* < 0.0001, *** *p* < 0.001, ** *p* < 0.01.

As compared to healthy skin, the levels of all investigated ceramides and glucosylceramide were higher in both non-lesional and lesional skin of AD patients (Figure 4), with exception of sphinganine ceramide, CER (dS), which reached a border-line significance (*p* = 0.09).

### 2.3. Association of Ceramide Markers with Disease Severity (SCORAD) and Skin Barrier Function (TEWL)

**Figure 5:**
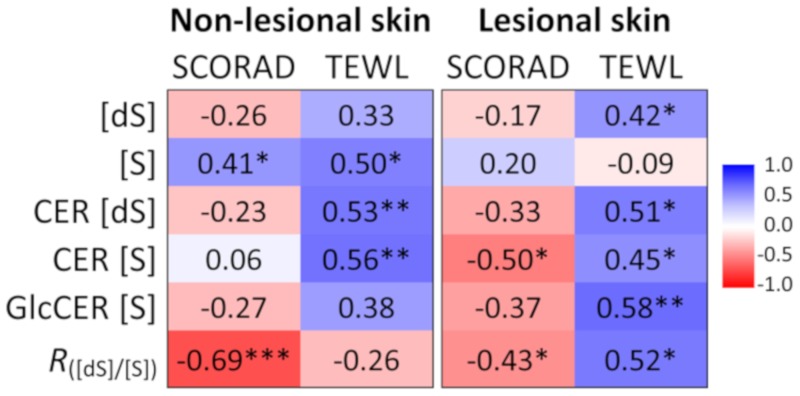
Spearman correlation coefficients between ceramide markers, atopic dermatitis severity (scoring atopic dermatitis, SCORAD) and trans-epidermal water loss (TEWL). (dS) = sphinganine, (S) = sphingosine, CER (dS) = sphinganine ceramides, CER (S) = sphingosine ceramides, GlcCER = glucosylceramide. R_[dS]/[S]_ = ratio between sphinganine and sphingosine. ***p < 0.001, **p < 0·01, *p < 0.05.

Spearman correlation analysis has been performed to compare individual ceramide markers with disease severity (scoring atopic dermatitis (SCORAD)) and skin barrier function (TEWL). Results are presented as a heat-map in Figure 5. The ratio between sphinganine and sphingosine was the only biomarker that showed a significant, inverse correlation with SCORAD in both, lesional and non-lesional skin (Figure 5). Furthermore, it showed the strongest association with SCORAD (*r* = ‒0.69, *p* < 0.001) of non-lesional skin in AD patients. Sphingosine was significantly correlated with SCORAD in non-lesional but not in lesional skin. Sphingosine-related ceramides, CER (S) were negatively correlated with SCORAD only in lesional skin. The majority of ceramide markers in both, non-lesional and lesional skin were positively correlated with TEWL (Figure 5).

**Figure 6:**
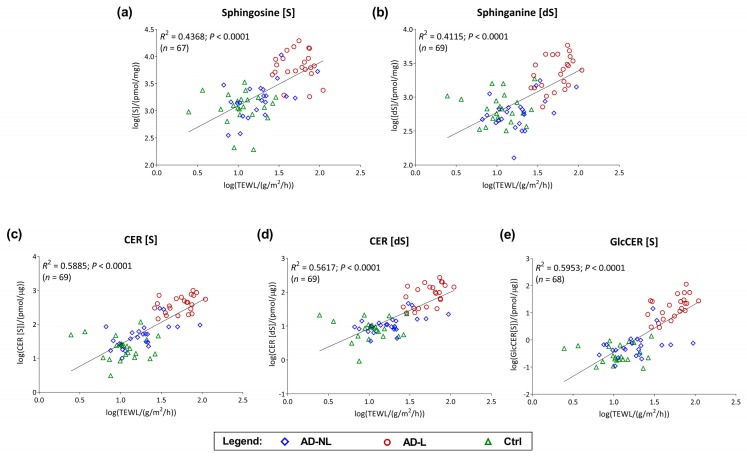
Linear regression analysis for the relationship between trans-epidermal water loss (TEWL) and ceramide markers (**a**) sphingosine, (S); (**b**) sphinganine, (dS); (**c**) CER, (S); (**d**) CER, (dS); and (**e**) glucosylceramide, GlcCER (S) in the SC of AD patients and healthy controls (Ctrl). NL = non-lesional skin; L = lesional skin. Data were log-transformed prior analysis. A *p*-value < 0.05 was considered significant.

A strong association between ceramide markers and skin barrier function was observed when data of healthy, AD non-lesional and lesional skin were pooled. As shown in Figure 6, all investigated markers showed a strong correlation with TEWL, with the coefficient of determination (*R*^2^) varying from 0.41 (sphinganine) to 0.60 (glucosylceramide).

### 2.4. Association of Lipid Biomarkers with Immunological Markers

**Figure 7:**
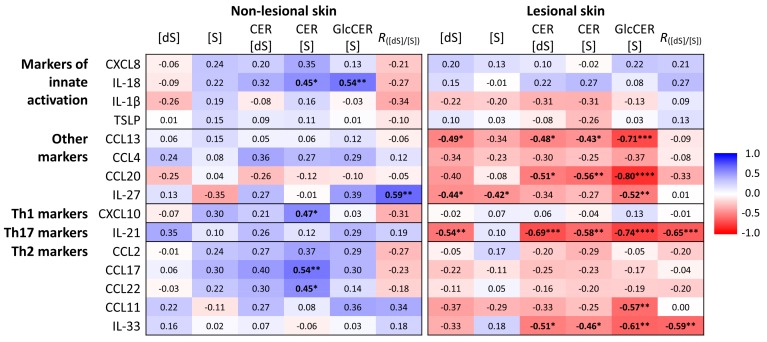
Spearman’s correlation coefficients between ceramide markers and cytokines. (S) = sphingosine, (dS) = sphinganine, CER (S) = sphingosine-related ceramides, CER (dS) = sphinganine-related ceramides, GlcCER (S) = glucosylceramide. **** *p* < 0.0001, *** *p* < 0.001, ** *p* < 0.01, * *p* < 0.05.

Next to disease severity, levels of investigated ceramide markers seem to be affected by the local cytokine milieu (Figure 7). The sphinganine/sphingosine ratio (*R*_[dS]/[S]_) was the only investigated marker that was correlated with at least one of the cytokines in both non-lesional and lesional skin. Interestingly, the cytokines that show significant association with ceramide markers are different in non-lesional and lesional skin, not only regarding the strength of association but also in direction. In lesional skin, all cytokines were inversely correlated, while in non-lesional skin all significant correlations were positive. The strongest correlation in lesional skin was found between glucosylceramides and IL-21, IL-33, CCL20, CCL11 and CCL13. In non-lesional skin, IL-18, CXCL10, CCL17 and CCL22 were significantly correlated with CER (S) and the sphinganine/sphingosine ratio with IL-27.

## 3. Discussion

In this study we showed that atopic skin has altered levels of free sphingosine and sphinganine and their corresponding ceramides, and that their profiles were related to skin barrier function, disease severity and local cytokine milieu. 

The levels of free sphingosine and sphinganine, the most abundant SBs in the SC showed increased levels in lesional skin compared with healthy controls. Despite of their relatively low SC levels (5–6% of total lipids) these SBs play an important role in antimicrobial defense, maintenance of the integrity of lipid lamellae and regulation of cell proliferation and differentiation, all of them known to be affected in AD [1,8,21,22,23,25]. Next to the absolute amount of SBs, we calculated their relative amount, which previously showed to affect lamellar organization in the SC [23]. The sphinganine/sphingosine ratio was reduced in AD skin, which is in line with the study of Loiseau et al. [23] performed in a mouse model of AD. Notably, in contrast to sphinganine and sphingosine levels, their ratio was significantly different from healthy skin also in clinically unaffected skin. Studies on the composition of free SBs in AD are scarce. Agrawal et al. [26] found increased levels of sphingosine in the sweat of AD patients, however contrary to these results Arikawa et al. [27] reported decreased sphingosine levels in the lesional and non-lesional skin of AD patients.

In addition to free sphingosine, also its related ceramides (CER (S)) and glucosylceramide showed elevated levels in AD skin. Although these changes were more pronounced in skin lesions, significantly altered levels were also observed in non-lesional skin. Sphinganine-related ceramide (CER (dS)) was increased only in lesional skin.

In the literature, sphingosine- and sphinganine-related CER were commonly measured as individual subclasses based on SB and acyl-chain composition, i.e., in the case of sphingosine these classes involve (NS), (AS) and (EOS) and for sphinganine (NdS), (AdS) and (EOdS) (Figure 1) [10,16,17]. In more recent studies, comprehensive analyses are performed in which tens of ceramide species containing FA’s with different chain lengths are measured within each of these subclasses. To our best knowledge, no information is available on the total amount of all CER-species sharing the same sphingoid base, therefore literature data for direct comparison are not available. However, several studies measured CER (NS) and (AS) which are the most abundant CER (S) (Figure 1) and therefore it may be assumed that these data are well comparable with results found in our study. Overall, the present findings are in accordance with previous studies, which reported increased CER (NS) and (AS) in AD skin of adults and in children [15,16,18,19,28,29]. 

To our best knowledge, this is the first study that shows increased levels of glucosylceramide in both, lesional and non-lesional skin of AD patients. Next to the de novo sphingolipid synthesis, CER are formed by degradation of glucosylceramides, which is mediated by lysosomal glucosylceramide-β-glucosidase (GBA) and from the breakdown of sphingomyelin catalyzed by acid sphingomylinase (aSMase) [14,17,24,30]. One of the possible explanations for elevated glucosylceramide levels in AD skin might be decreased activity or expression of GBA. Previously, altered expression of GBA has been reported in AD skin [15]. Increased levels of glucosylceramides might also have been affected by decreased activity of GBA, a pH-dependent enzyme [15]. In the present study lesional skin showed increased pH, which showed to lead to lower activity of GBA [15].

A further novel finding in the present study is a significant correlation between the levels of certain ceramide markers and immunological mediators, revealing the interplay between immune response and ceramide composition in AD. Strikingly, the associations of ceramide markers with cytokines differed between unaffected and lesional skin, not only in the type of cytokine and strength of association but also in direction. In lesional skin, all cytokines that showed significant association were inversely correlated with ceramide markers, while in unaffected skin a positive correlation was found. Different cytokine milieu in lesional and non-lesional skin has previously been reported and is likely caused by the recruitment of specific immune cells and secreted cytokines to the lesions [2,5]. In the present study, the strongest association with ceramide markers in lesional skin was found for the type I cytokine family members IL-21 and IL-33, and chemoattractants CCL11 (eotaxin-1), CCL13 and CCL20, all of them previously shown to be predominantly present in lesional AD skin [31,32,33]. In non-lesional skin, the strongest correlation with investigated ceramide markers was found for Th2 chemokines CCL17 (TARC) and CCL22 (MDC), which are key cytokines in AD pathophysiology [34]. Th-2 cytokines have previously been shown to be associated with relative distribution of ceramides with respect to the total chain length [19]. In the non-lesional skin, we also found a correlation of CER (S) with IL-18, a representative of the innate immune system and furthermore with CXCL10, the only Th1 cytokine investigated in this study. The effect of cytokines on ceramide profiles has mainly been investigated in in vitro or mouse models of AD [35,36,37,38].

One of the objectives of this study was to explore the suitability of ceramide biomarkers to assess severity of disease and skin barrier function. Interestingly, the ratio between sphinganine over sphingosine showed the strongest association with SCORAD in both, clinically unaffected and lesional skin. The majority of investigated ceramide markers showed a significant correlation with TEWL, revealing their importance for the skin barrier function. 

A novel analysis method demonstrated to be a robust and simple approach. So far, there are more than several hundreds of structurally different CERs and additional ceramide subclasses are still being identified [14,17]. This poses a major challenge as quantification of individual lipid species by LC–MS/MS requires standards that behave similarly with respect to response in the mass spectrometer and for most ceramide species these are not available [39]. Often, methods are semi-quantitative, provide only a relative amount of an individual CER and are based on a single standard for all CER species [40,41]. The advantage of our approach is that all ceramides which share the same SB are measured together after removal of FA chain. This makes analytical determination more sensitive but also accurate as there is limited bias due to differences in the detector response. However, we acknowledge that this approach has limitations. Synthesis of ceramides is complex and involves a large number of pathways and enzymes which are specific with respect to SB and acyl-chain preference [14]. Therefore, our approach does not provide mechanistic insight in metabolic pathways as compared to detailed lipidomic analysis [39,42,43]. Nevertheless, the investigated ceramide markers showed good correlation with skin barrier function and disease severity, and the strength of association is not inferior to that reported in studies based on more comprehensive approaches [17]. Important in the context of biomarkers field is the association that was found between investigated ceramide markers and key cytokines in AD. Recently, new therapies have been introduced, which target various immune pathways in AD, e.g., dupilimab, the first approved immune therapy for AD which specifically targets the Th2-immune pathway [44]. In the present study we showed that the levels of several Th2 cytokines are correlated with those of the investigated ceramide markers. In the light of therapy response, it would be interesting to investigate the levels of the ceramide markers in future clinical trials.

To summarize, we found difference in the levels of SBs as well as their ceramides between healthy and AD skin. The finding that alterations were more prominent in lesional skin and the observed association with disease severity, suggest that the immune/inflammatory response plays an important role in metabolism of ceramides in AD. In non-lesional skin several ceramide markers were shown to be also altered, implying that changes in ceramide composition are already present in skin, which appears to be normal. Further studies are needed to investigate whether these ceramide components might be useful in the clinics e.g., as early effect biomarkers in non-lesional skin or for the monitoring of (immune) therapy.

## 4. Materials and Methods 

### 4.1. Study Population

We included 25 Caucasian patients with AD (19 females and 6 males; median age, 24 years; range, 19–38 years) and 23 healthy controls (15 females and 8 males; median age, 32 years; range, 22–45 years). Patients with moderate to severe AD (SCORAD value mean 55.8, range 33.8-81.1) were recruited at the University Department of Dermatovenereology of Clinical Hospital Centre Zagreb and School of Medicine of University of Zagreb, Croatia. Diagnosis of AD was confirmed by experienced dermatologists working in the Outpatient allergology department using AD diagnostic criteria by Hanifin and Rajka [45]. The severity of the disease was assessed by SCORAD index of the European Task force on Atopic dermatitis [46] by the same dermatologists during the entire study. Nine patients had moderate AD defined by SCORAD between 15 and 50 points (mean SCORAD value 42.2, range 33.8–48.9) and sixteen patients had severe AD defined by SCORAD ≥ 50 (mean SCORAD value 63.4, range 51.4–81.1). Atopic diathesis was positive in 16 patients, allergic asthma in 10 patients, allergic rhinitis in 9 patients and allergic conjunctivitis in 3 patients. Exclusion criteria for patients were systemic therapy with immunosuppressants or phototherapy within 4 weeks prior the study, use of topical steroids or immunomodulators in the previous week, or moisturizers within 12 h prior evaluation. 

The control group of participants consisted of 23 healthy individuals with no visible skin damage and no (history of) past or present inflammatory skin diseases. All healthy individuals underwent a clinical examination in order to exclude milder, unrecognized forms of AD. Exclusion criteria for control group were inflammatory skin disorders, systemic disorders and personal anamnestic data on atopic disorders or any kind of eczema in childhood. Healthy participants were instructed not to use emolients, soap, perfumes or any other cosmetics or creams on the forearm skin within 24 h prior evaluation. 

All individuals who participated in the study provided written informed consent, which complied with the principles of the Declaration of Helsinki. This study was approved by the Medical Ethical Committees of the University Hospital Centre Zagreb and School of Medicine, University of Zagreb (27^th^ June 2014, 02/21/JG). Written informed consent was obtained from all subjects prior to the study. 

### 4.2. Sampling of the Stratum Corneum

**Figure 8:**
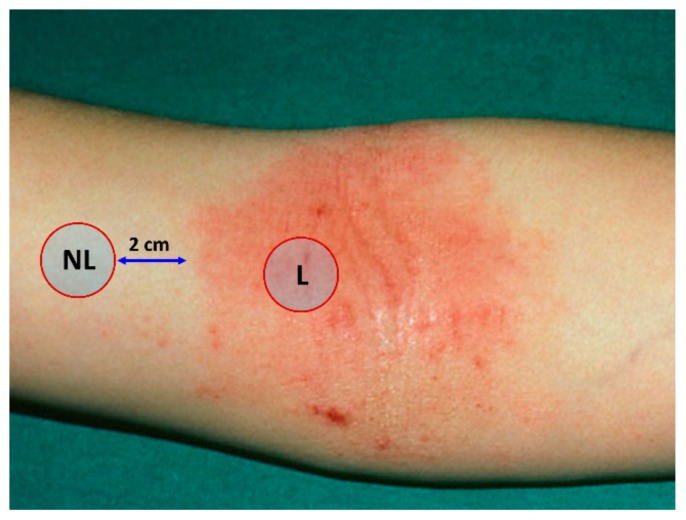
Lesional (L) and non-lesional (NL) skin sites for the stratum corneum collection.

The SC was sampled using the previously described method [47]. We placed circular adhesive tapes (22 mm diameter, 3.8 cm^2^, D-Squame Discs; Monaderm, Monaco, France) on lesional and non-lesional (at a distance of 2 cm from the lesion) forearm skin of patients and of healthy controls (Figure 8). Adhesive tapes were pressed for 10 s with a pressure of 225 g cm^2^, using a D-Squame Pressure Instrument D500 (CuDerm Corporation, Dallas, TX, USA). Tape strips were gently removed with tweezers and stored individually in 2 mL cryo-vials. Sequentially, eight consecutive tape strips were collected from the same skin site and immediately stored at –20 °C. For the analysis, the 4^th^ tape was used, and for cytokine analysis 6^th^ tape.

### 4.3. Measurements of TEWL And pH

TEWL was measured with the open chamber system and skin pH was assessed by a pH 905 probe using a Tewameter TM300 (Courage + Khazaka electronic, Köln, Germany). Twenty minutes prior to the application, the subjects rested with their sleeves rolled-up in the examination room, where the temperature was 20–21°C and the relative humidity ranged between 50% and 60%.

### 4.4. Ceramide Analysis

**Figure 9:**
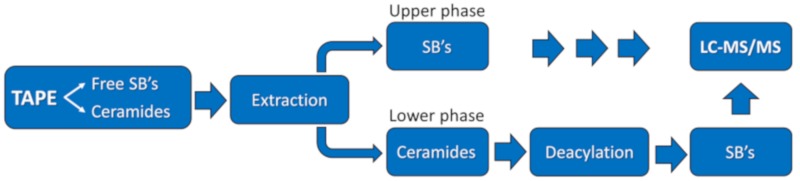
Scheme for determination of SBs and ceramides in the stratum corneum tapes.

Free sphingoid bases and their ceramides were determined in a 2-step procedure (Figure 9). In the first step, free sphingoid bases were separated from ceramides by extraction with methanol:chloroform:water. The lower phase contains ceramides and the upper phase free sphingoid bases. The ceramides in the lower phase were subsequently deacylated to corresponding sphingoid bases by use of microwave-assisted hydrolysis in methanolic NaOH, according to the procedure described elsewhere [48]. Free sphingoid bases and the sphingoid bases derived after deacylation of ceramides were determined in separate runs by LC-MS/MS as described in details elsewhere [49]. Details on the extraction procedure are shown below. 

#### 4.4.1. Chemicals

Sphingosine (d18:1), d7-sphingosine (d18:1), sphinganine (d18:0), d7-sphinganine (d18:0), glucosylsphingosine (d18:1), and d5-glucosylsphingosine (d18:1), were purchased from Avanti Polar Lipids Inc. and all organic solvents were from Biosolve (LC-MS/MS quality). Formic acid, butanol, and hydrochloric acid were obtained from Merck and sodium hydroxide and ammonium formate from Sigma (Alabaster, AL, USA).

#### 4.4.2. Extraction from the Tapes and Processing

Ceramides including glucosylceramide and sphingoid bases were extracted from the tapes by the addition of 1 mL of methanol:chloroform:water, 12:6:1, v/v/v. The extract was dried under a stream of nitrogen at 40 °C and separation of phases was induced by the addition of methanol:chloroform:100 mM ammonium formate in 20% formic acid, 1:1:1, v/v/v). The lower phase, containing ceramides, was dried under a stream of nitrogen and the residue was dissolved in 500 µL 0.1 M sodium hydroxide in methanol. Next, samples were deacylated (microwave-assisted) and internal standards (d7-sphingosine (d18:1), d7-sphinganine (d18:0) and d5-glucosylsphingosine (d18:1) were added to each sample), followed by evaporation of methanol (N2, 40 °C). Thereafter, samples were subjected to extraction with butanol:water (1:1, v/v). To the upper phase, internal standards were added (d7-sphingosine (d18:1) and d7-sphinganine (d18:0)), samples were dried (N2, 40 °C) and butanol:water (1:1, v/v) was added. After drying the butanol phase, samples were dissolved in 120 µL methanol and analyzed by LC–MS/MS. Levels of ceramides and sphingoid bases were calculated using calibration lines within the appropriate concentration range, according to the internal standard ratio method.

#### 4.4.3. LC–MS/MS Analysis

Sphingoid bases were separated by RP-UPLC using an Acquity I-Class UPLC with BEH C18 column, 2.1 × 50 mm with 1.7 μm particle size (Waters Inc.) and detected by electron spray ionisation in positive mode and MS/MS-instrument (Xevo TQ MS, Waters Inc., Milford, MA, U.S.A.) in multiple reaction monitoring (MRM) mode (see Supplementary Table S1) according to the method described previously [49]. Details on MS analysis are shown in Supplementary Table S1. 

To compensate for variable amount of stratum corneum on the tape, the concentrations of all analytes were normalized for protein content on the tape [47]. For that purpose, 150 µl of the extract obtained after the extraction with methanol:chloroform:water was evaporated to dryness under a stream of nitrogen, and to the residue 250 µl water was added. The protein amount was determined by Pierce Micro BCA protein assay (Thermo Fisher Scientific, Rockford, IL, USA).

### 4.5. Cytokine Analysis

Cytokines were determined according to the procedure described in detail elsewhere [47]. Briefly, extraction of cytokines and soluble proteins was performed with an ultrasound sonifier equipped with a probe (Salm and Kipp, Breukelen, the Netherlands) for 15 min in ice water with 1.2 mL phosphate-buffered saline (Merck, Darmstadt, Germany) containing 0.005 % Tween 20 (Sigma-Aldrich, Zwijndrecht, the Netherlands). In total, concentrations of 21 cytokines were measured on preconfigured multiplex panels using MESO QuickPlex SQ 120 (MSD, Rockville, MA, USA) according to the manufacturer’s instructions. 

For statistical analysis, cytokine concentrations below the detection limit (but above the bottom of the curve) or above the detection limit were taken unchanged, and cytokine concentrations that were below the fit curve range (signal below the bottom of the bottom-of-the-curve fit, no concentration given) were assigned half the value of the lowest sample concentration below the detection limit to maintain the ranking order. Due to a high number of samples with concentration values below the detection limit (more than 30% of samples), CCL3, CCL26, IL-17A, IL-22, IL-23 and IL-33 were excluded from further statistical analysis.

To compensate for variable amount of stratum corneum on the tape, the concentrations of all cytokines were normalized for protein content determined in the tape extracts using Pierce Micro BCA protein assay (Thermo Fisher Scientific, Rockford, IL, USA), as described elsewhere [47]. 

### 4.6. Statistical Analysis 

For sample size calculation, we have used data from our previous study on CER (NS) levels in non-lesional SC of AD patients and control subjects [17]. We calculated that 19 subjects per group would be required to detect significant differences in levels of investigated markers between two groups with a 95% confidence interval and 80% power [50].

Calculations were performed by using Prism 8 software (GraphPad, San Diego, CA, USA). Distribution of data was tested using the Shapiro–Wilk normality test. Differences in the levels of all parameters between healthy controls and AD skin were determined by two-tailed Welch’s *t*-test or two-tailed Mann–Whitney test, dependently on data distribution. Correlation analysis between lipid markers, SCORAD, cytokine levels and TEWL was performed by Spearman’s correlation. Due to explorative character of the study obtained *p*-values were not corrected for multiple testing. Before correlation and regression analysis, data were log-transformed.

## Supplementary Materials

Supplementary Table S1 can be found at https://www.mdpi.com/1422-0067/21/6/1958/s1.

## Abbreviations

AD	atopic dermatitis
aSMase	acid sphingomylinase
CER CER (S) CER (dS)	ceramides d18:1 ceramides d18:0 ceramides
FA FFA GBA GBA1 GlcCER LC MS (S) (dS) SB SC SCORAD TEWL	fatty acid free fatty acid β-glucosylcerebrosidase lysosomal glucosylceramide-β-glucosidase glucosyl ceramide liquid chromatography mass spectrometry sphingosine (d18:1) sphinganine (d18:0) sphingoid base stratum corneum scoring atopic dermatitis trans-epidermal water loss
