# Supplementary material for: Altered Levels of Sphingosine, Sphinganine and Their Ceramides in Atopic Dermatitis Are Related to Skin Barrier Function, Disease Severity and Local Cytokine Milieu
Source: Int J Mol Sci. 2020 Mar 13;21(6):1958. doi: 10.3390/ijms21061958 (PMC7139865; doi:10.3390/ijms21061958)
Supplement: Supplementary file 1 [file ijms-21-01958-s001.pdf]

SUPPLEMENTARY FILE

Altered Levels of Sphingosine, Sphinganine and Their Ceramides in Atopic Dermatitis Are Related to Skin Barrier Function, Disease Severity and Local Cytokine Milieu

Ruzica Jurakic Toncic [1], Ivone Jakasa [2,3], Suzana Ljubojevic Hadzavdic [1], Susan MI Goorden [4], Karen JM Ghauharali-van der Vlugt [4], Femke S Stet [4], Anamaria Balic [1], Mikela Petkovic [1], Borna Pavicic [1], Kristina Zuzul [1], Branka Marinovic [1] and Sanja Kezic [3,*]

1 Department of Dermatology and Venereology, University Hospital Center, Zagreb and University of Zagreb School of Medicine, 10000 Zagreb, Croatia; rjtoncic@gmail.com (R.J.T.); suzana.ljubojevic@gmail.com (S.L.H.); jovicanamaria@gmail.com (A.B.); dr.mikela@gmail.com (M.P.); borna.pavicic@gmail.com (B.P.); zuzulkristina@gmail.com (K.Z.); branka.marinovic@kbc-zagreb.hr (B.M.)

2 Laboratory for Analytical Chemistry, Department of Chemistry and Biochemistry, Faculty of Food Technology and Biotechnology, University of Zagreb, 10000 Zagreb, Croatia; ijakasa@pbf.unizg.hr and/or i.jakasa@amsterdamumc.nl (I.J.)

3 Amsterdam UMC, University of Amsterdam, Coronel Institute of Occupational Health, Amsterdam Public Health research institute, 1105 AZ Amsterdam, Netherlands, i.jakasa@amsterdamumc.nl (I.J.); s.kezic@amsterdamumc.nl (S.K.)

4 Laboratory Genetic Metabolic Disease, Amsterdam UMC, 1105 AZ Amsterdam, Netherlands; s.m.goorden@amsterdamumc.nl (S.M.I.G.); j.m.ghauharali@amsterdamumc.nl (K.J.M.G.V.); f.s.stet@amsterdamumc.nl (F.S.S.)

* Correspondence: s.kezic@amsterdamumc.nl

Supplementary Table 1 is related to section 4. Methods and materials/ 4.4. Ceramide analysis/4.4.3. LC-MS/MS analysis.

Supplementary Table 1. Details on LC-MS/MS analysis of sphingoid bases and their ceramides. * MRM: Multiple Reaction Monitoring.

Compound	MRM* (m/z)	Cone voltage (V)	Collision voltage (V)	Retention time (min)	Calibration curve	Internal standard
Sphinganine (d18:0)	302.3 > 284.3	25	15	3.11	Sphinganine (d18:0)	d7-Sphinganine (d18:0)
Sphingosine (d18:1)	300.3 > 282.3	20	10	3.07	Sphingosine (d18:1)	d7-Sphingosine (d18:1)
Glucosylsphingosine (d18:1)	462.4 > 282.3	25	20	3.01	Glucosylsphingosine (d18:1)	d5-Glucosylsphingosine (d18:1)
d7-Sphinganine (d18:0)	309.3 > 291.3	25	15	3.11	Not applicable	Not applicable
d7-Sphingosine (d18:1)	307.3 > 289.3	20	10	3.07	Not applicable	Not applicable
d5-Glucosylsphingosine (d18:1)	467.4 > 287.4	25	20	3.01	Not applicable	Not applicable
